# Synthesis of phosphatidylcholine in rats with oleic acid-induced pulmonary edema and effect of exogenous pulmonary surfactant on its *De Novo* synthesis

**DOI:** 10.1371/journal.pone.0193719

**Published:** 2018-03-19

**Authors:** Xiwen Gao, Peiyu Qian, Dong Cen, Weijun Hong, Qing Peng, Min Xue

**Affiliations:** 1 Department of Respiratory Diseases, Minhang Hospital, Fudan University, Minhang District, Shanghai, P.R. China; 2 Oncology Bioinformatic Research Center, Minhang Hospital, Fudan University, Minhang District, Shanghai, P.R. China; 3 Centre for Clinical Laboratory, Ningbo Yinzhou No 2 Hospital, Zhejiang; National Yang-Ming University, TAIWAN

## Abstract

In mammals, oleic acid (OA) induces pulmonary edema (PE), which can initiate acute lung injury (ALI) and lead to acute respiratory distress syndrome (ARDS). Pulmonary surfactant (PS) plays a key role in a broad range of treatments for ARDS. The aim of the present investigation was to assess changes in the synthesis of phosphatidylcholine (PC) from choline and determine the effect of exogenous PS on its *de novo* synthesis in rats with OA-induced PE. Experimental rats were randomized into three groups, including a control group, OA-induced PE group, and OA-induced group treated with exogenous PS (OA-PS). Twenty-four rats were sacrificed 4 h after induction of the OA model, and tissue was examined by light and electron microscopy to assess the severity of ALI using an established scoring system at the end of the experiment. After 15 μCi ^3^H-choline chloride was injected intravenously, eight rats in each group were sacrificed at 4, 8, and 16 h. The radioactivity of ^3^H incorporated into total phospholipid (TPL) and desaturated phosphatidylcholine (DSPC) was measured in bronchoalveolar lavage fluid (BALF) and lung tissue (LT) using a liquid scintillation counter and was expressed as counts per minute (CPM). Results showed that TPL, DSPC, and the ratio of DSPC/total protein (TP) in lung tissue decreased 4 h after challenge with OA, but the levels recovered after 8 and 16 h. At 8 h after injection, ^3^H-TPL and ^3^H-DSPC radioactivity in the lungs reached its peak. Importantly, ^3^H-DSPC CPM were significantly lower in the PS treatment group (LT: Control: 62327 ± 9108; OA-PE: 97315 ± 10083; OA-PS: 45127 ± 10034, *P* < 0.05; BALF: Control: 7771 ± 1768; OA-PE: 8097 ± 1799; OA-PE: 3651 ± 1027, *P* < 0.05). Furthermore, DSPC secretory rate (SR) in the lungs was significantly lower in the PS treatment group at 4 h after injection (Control: 0.014 ± 0.003; OA-PE: 0.011 ± 0.004; OA-PS: 0.023 ± 0.006, *P* < 0.05). Therefore, we hypothesize that exogenous PS treatments may adversely affect endogenous *de novo* synthetic and secretory phospholipid pathways via feedback inhibition. This novel finding reveals the specific involvement of exogenous PS in endogenous synthetic and secretory phospholipid pathways during the treatment of ARDS. This information improves our understanding of how PS treatment is beneficial against ARDS and opens new opportunities for expanding its use.

## Introduction

In 1967, acute lung injury (ALI) was first proposed and defined by Petty et al [1]. Recently, there have been significant advances in understanding its pathogenesis and pathophysiology. ALI and acute respiratory distress syndrome (ARDS) are thought to involve a cascade of damage induced by direct pulmonary injury or inflammatory responses that may inhibit the dispersion of oxygen and contribute to lung dysfunction [2]. Accordingly, patients may die of respiratory failure, and the mortality rate may reach up to 45% [3]. There is still no effective pharmacotherapy for ARDS and the mortality remains high. The development of technology made new breakthrough such as mesenchymal stem (stromal) cells treatments[4]. However, the effectiveness of treatment still in evaluation. Although numerous treatment strategies have been proposed, results suggest that ALI or ARDS patients require administration of pulmonary surfactant (PS), positive end-expiratory pressure (PEEP), and circulation support at an early stage to prevent alveolar collapse. Nevertheless, the prognosis for patients remains poor [5].

In mammals, oleic acid (OA) induces pulmonary edema (PE), which can initiate ALI and further lead to ARDS. Different models implicate different mechanisms of ARDS, including damage to the vascular barrier function and dysfunction of the surfactant system [6]. In OA-induced PE (where OA is injected into a central vein), the flooding of alveoli leads to PS dysfunction whereas disruption of the capillary alveolar membrane aggravates capillary leakage [7]. This mimics the pathological mechanisms of ARDS, producing acute endothelial and alveolar epithelial cell necrosis, resulting in multiple pulmonary microembolisms and protein-rich pulmonary edemas in a perfusion distribution-dependent manner [8].

PS plays a key role in a broad range of treatments for ARDS. Moreover, its administration is effective and safe. Under physiological conditions, the special structure of PS can reduce alveolar surface tension and restore its normal functioning, thereby maintaining alveolar cavity morphology [9]. More than 90% of PS is made of phospholipids and about 1% is contributed by hydrophobic protein. Reuptake of degradative phosphatidylcholine (PC) is the main pathway for the synthesis of endogenous PS, whereas the remaining 10–14% of PS is synthesized through *de novo* pathways [10]. Studies in rats have shown a reduction in PS on the surface of the alveolar cavity following intravenous OA injections. However, whether the *de novo* synthesis of PS is inhibited or promoted after exogenous PS therapy in rats with OA-induced lung injury remains unknown.

Therefore, in this study we assessed the pathophysiology of lung tissue and the synthesis of PC from choline using an OA-induced lung injury model in rats. The radioactivity of ^3^H incorporated into total phospholipid (TPL) and desaturated phosphatidylcholine (DSPC) in bronchoalveolar lavage fluid (BALF) and lung tissue (LT) was assessed to determine the effect of exogenous PS on the *de novo* synthesis of endogenous PS. Tissue was examined by light and electron microscopy to assess the progression of ALI with an established scoring system at the end of the experiment. For this assessment, we compared OA-induced changes in the synthesis of PS in rats treated with or without porcine pulmonary surfactant (PPS).

This study is important as it reveals the specific involvement of exogenous PS in endogenous *de novo* synthetic and secretory phospholipid pathways. Improved understanding of this mechanism is instrumental for improving ARDS treatment.

## Materials and methods

Use of animals and animal tissues in laboratory trials of ARDS treatments was approved by the State Food and Drug Administration of China and the ethical board of Minhang Hospital, Fudan University.

### Surfactant preparation

Fresh Pig BALF was collected from the local animal farm and was prepared using normal saline. PPS (PS emulsion 40 mg/mL; Pediatric Research Institute, Fudan University) was extracted from BALF using sequential centrifugation, chloroform/methanol (2:1, V/V) extraction, and acetone precipitation to remove neutral fat. After being dried and weighed, PPS was suspended in saline solution with phospholipid (40 mg/mL) and stored at 4°C [11].

### Animals

More than 100 healthy male Sprague-Dawley rats (200–250 g each) were provided by the Shanghai Laboratory Animal Center, Chinese Academy of Sciences. All rats were housed at controlled temperature (23°C to 25°C) and lighting (8:00 AM to 8:00 PM light, 8:00 PM to 8:00 AM dark). Each rat was supplied with standard food and sterile drinking water. Based on the records by the animal housing keeper, no signs of ill health (such as low activity, wounds caused by fights with other rats, or abnormal hair loss) could be observed before OA treatment. Additionally, no abnormal rat death was observed during the course of the experiment. Each rat was sacrificed by anesthesia with 10% chloral hydrate (0.4 mL/100 g) following abdominal aorta bleeding, in line with institutional guidelines of Fudan University for health and care of experimental animals.

Notes: The ethical statement regarding the use of 10% chloral hydrate (0.4 mL/100 g) following abdominal aorta bleeding was approved by Minhang Hospital, Fudan University, and is attached to the supporting information. Chloral hydrate is not an acceptable method of euthanasia according to AVMA guidelines [12]. Indeed, chloral hydrate-induced hypoxemia may compromise the accuracy of protein and mRNA levels of an activity-related protein. Whereas other agents or methods for sacrificing the animals could be more suitable for both surgical anesthesia and euthanasia, experimental design was limited by local laboratory facilities and the study’s aims. To this end, the use of chloral hydrate had no bearing on our understanding of the synthesis of PC in rats with OA-induced PE. Moreover, hypoxemia and respiratory distress caused by chloral hydrate injection, could result also from other forms of euthanasia, such as dislocation of the vertebrae and asphyxia by CO_2_. Based on other reports, chloral hydrate may have potential side effects on the nervous system, however this aspect was not investigated in the present study.

### Allocation of rat groups

Rats were randomized into three groups, including a control group (n = 38), OA-induced PE (OA-PE) group (n = 54), and exogenous PS (OA-PS) treatment group (n = 30). OA (0.06 mL/kg; Sinopharm Chemical Reagent Co. Ltd., China) was injected into the central vein in both OA-PE and OA-PS animals. After the OA-PE model was created, tracheal instillation of PPS (50 mg/kg) was administered to the OA-PS group. All rats used in the experiment stayed in the animal house under specific-pathogen-free conditions, and were supplied with clear water and food. A health recorded was compiled daily.

### Histopathology of an acute pulmonary edema model

Six rats from each group were sacrificed at 4 h after OA-induced PE, and the lungs were harvested for histopathological hematoxylin-eosin (H-E) staining. Tissue was examined by light and electron microscopy to assess the extent of ALI using an established scoring system at the end of the experiment. Lung injury was scored in a blinded fashion. Then, lung tissue was harvested using a Coulter counter (Coulter Electronics, USA), a cytocentrifuged preparation (cytosine 2; Shandon Southern Products, UK) of BALF was stained with Wright-Giemsa, and cell differentiation was assessed. Hyperemia, atelectasis, and neutrophil infiltration were scored as: 0 = minimal; 1 = mild; 2 = moderate; 3 = severe; 4 = maximal. Intra-alveolar edema was scored as: 0 = absent; 1 = present.

### Preparation and infusion of radiolabels

As a synthetic precursor for PC, 15 μCi ^3^H-methyl choline chloride ([^3^H]-labeled choline chloride, 84 Ci/mmol; Amersham Pharmacia Biotech, USA) was added to normal saline (NS, 0.4 mL) to prepare the radiolabels, 5 μL mixed liquid was pumped back into a liquid scintillation counter (LS 6500, Beckman, UK), and dried for analysis. Further analysis of PC metabolism in LT and BALF, including ^3^H-TPL, ^3^H-DSPC, and secretory rates, was conducted to assess the synthesis of PS. After construction of the OA model, ^3^H-choline chloride [^3^H-choline chloride and NS mixed liquid, 0.2 mL] was gently injected intravenously (> 2 min). The NS was then re-extracted (0.1 mL) and the syringe was washed twice.

### Surfactant phospholipid analysis in LT and BALF

Eight rats in each group were sacrificed at 4, 8, and 16 h. The radioactivity of ^3^H incorporated into TPL and DSPC was measured in BALF and LT after lavage. The size of the pool of PC was also measured in both BALF and LT. To this end, we opened the abdominal cavity of each rat, and the right ventricle was perfused with NS (15 mL) at 5 mL/min. Bronchoalveolar lavage was performed at 2°C with three separate volumes of 8 mL NS, with each volume being instilled and withdrawn three times. The resulting BALF was centrifuged at 200 × *g* at 4°C for 10 min. Cells were discarded, and the supernatant (8 mL) was weighed and incubated at -20°C. Bronchoalveolar lavage was repeated twice with 8 mL of NS, BALF was discarded, lungs were separated, and small tissue fragments cut from the lungs (5 mL) were kept in preservation solution (10 mL methanol plus 6 mL NS) at -20°C.

**Fig 1:**
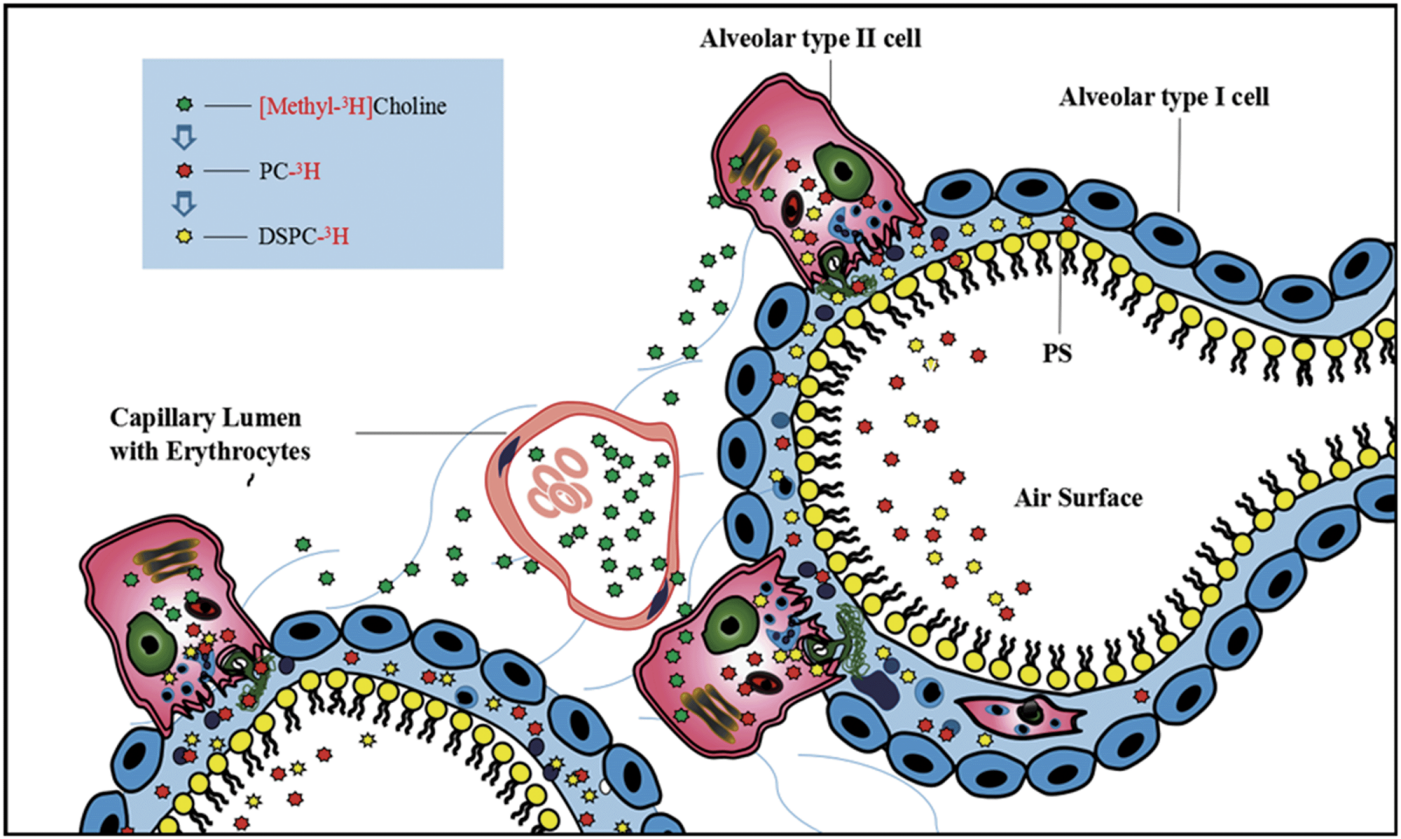
Alveolar structure and schematic design of isotope tracing. ^3^H-labeled methyl chloride choline is involved in surfactant phospholipid synthesis of alveolar type II cells. The level of ^3^H in phospholipids was monitored to detect the amount originating from endogenous phospholipid synthesis. The radioactivity of TPL and DSPC in BALF and LT indicates the total content of ^3^H-labeled choline chloride incorporated into TPL and DSPC, which reflects the newly synthesized TPL and DSPC and the body’s ability to synthesize PC. The secretory rate of TPL was expressed as the ratio of radioactivity in BALF and the whole lung (BALF+LT). The secretory rates of TPL and DSPC indicate the ability of alveolar type II epithelial cells to secrete PC into the alveolar space.

Air-dried samples were treated with 5 mL liquid scintillator toluene solution [0.03% POPOP, Fluka Switzerland; 0.5% 2,5-diphenyloxazoline (PPO), Shanghai Chemical Reagent Company, Sinopharm]. Radiolabels were counted with an LS 6500 liquid scintillation counter and expressed as CPM. The ^3^H isotope tracer technique involving methyl chloride [13] is shown in Fig 1.

### Phospholipid extraction

For the extraction of phospholipids, 20 mL chloroform was added to a lung tissue mixture (volume of specimen: methanol: chloroform = 1:1:2), centrifuged at 7000 rpm for 10 s at 10-s intervals, and frozen overnight. The same volume of methanol and a two-fold volume of chloroform was added to each BALF sample (8 mL), with samples then undergoing constant oscillation for 10 min before being frozen overnight.

### ^3^H-TPL and ^3^H-DSPC measurement in LT and BALF

H_3TPLCPMLT=cpm×VLTVassay=cpm×5ml×41ml

H_3DSPCCPMLT=cpm×VLTVassay=cpm×5ml×4×0.9ml2ml×0.75ml

H_3TPLCPMBALF=cpm×VBALFVassay=cpm×8ml×45ml

H_3DSPCCPMBALF=cpm×VBALFVassay=cpm×8ml×4×0.9ml6ml×0.6ml

^3^H-TPL in BALF was measured with 5 mL BALF dissolved in chloroform (V_assay_). For ^3^H-DSPC measurement, 6 mL BALF dissolved in chloroform dried with nitrogen gas and mixed with osmium tetroxide solution (0.5g/ml) to flow through the aluminum column. The elution (chloroform: methanol: ammonia = 70:30:2) was dried out and re-dissolved with 0.9 mL chloroform. Finally, 0.6 mL was collected for ^3^H-DSPC measurement. ^3^H-TPL in LT was measured with 1 mL lung tissue homogenization (LTH) buffer dissolved in chloroform. Again, 2 ml LTH containing RNase and DNase was treated with aluminum chromatography, and the elution was dissolved with 0.9 mL chloroform. Finally, 0.75 mL was collected for ^3^H-DSPC in LT measurement. Total pulse number in LT or BALF was expressed as CPM. V_LT_ or V_BALF_ was the volume of LT after dilution in chloroform and methanol. V_assay_ was the volume of LT or BALF used for the assay.

### Statistical analysis

Data are expressed as mean ± standard deviation (SD). Differences before and after induction of lung injury were analyzed by a paired *t*-test. Differences in CPM levels among groups and sacrifice times (4, 8, and 16 h) were calculated using a one-way ANOVA combined with post hoc multiple comparisons by Bonferroni’s correction. Differences between both lung injury models were analyzed by the Student-Newman-Keuls test. For all test procedures, *P* < 0.05 was considered significant (SPSS Inc., USA).

## Results

### General condition of rats

**Fig 2:**
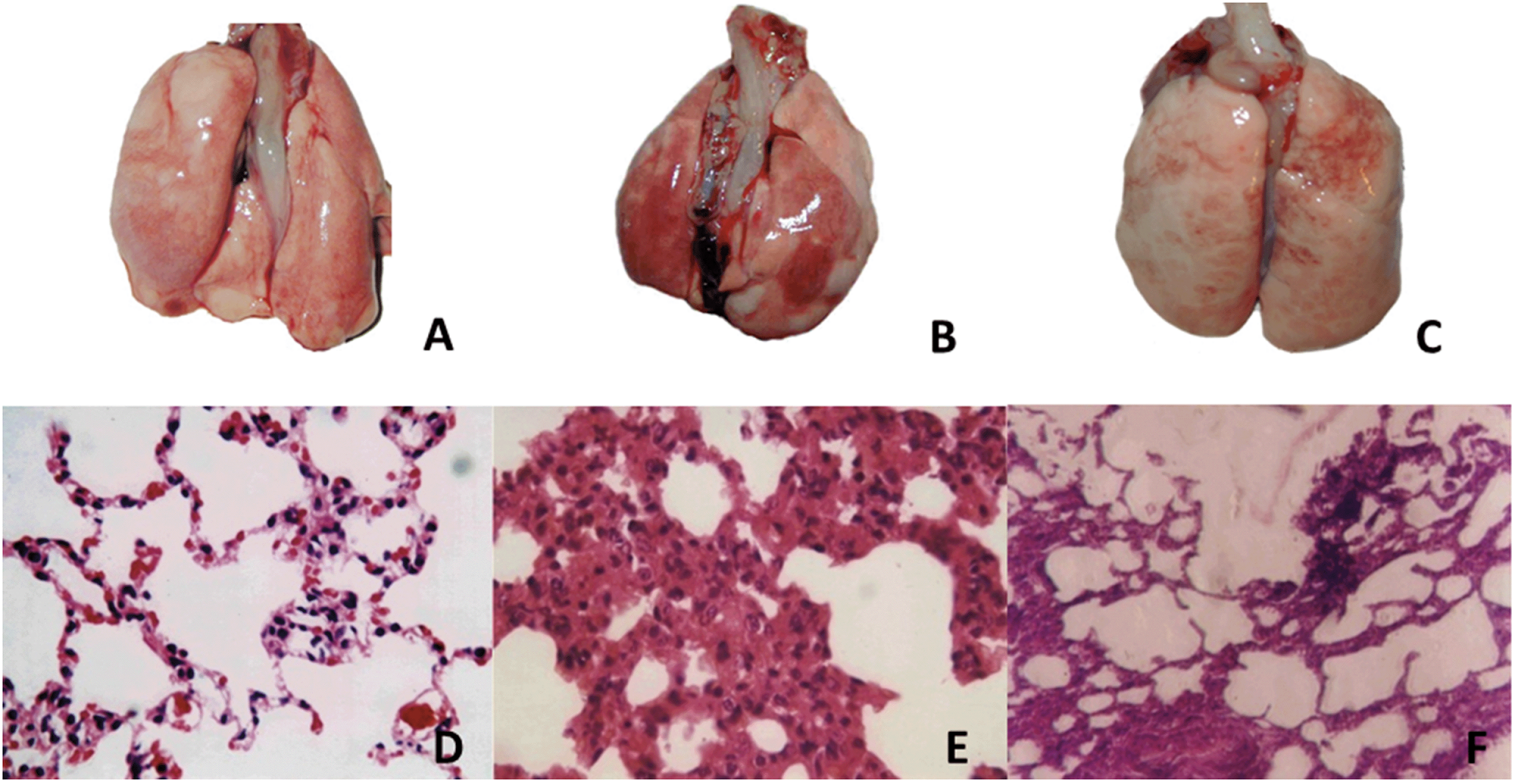
Histopathological characterization in rats as determined by gross morphology and hematoxylin-eosin (H-E) staining (×400). A and D: Normal control group; B and E: OA-PE group; C and F: OA-PS treatment group. A, B, C: gross morphology; D, E, F: H-E staining.

Animal body weight was similar across the three groups. Injection of OA did not cause severe death before sacrificing the animals. OA injection induced atelectasis, edema, and hemorrhage, as seen by comparing gross morphology of lung sections in control (Fig 2A) and OA treatment (Fig 2B and 2C) groups. Notably, symptoms in the OA-PS (Fig 2C) group were milder than those in the OA-induced PE group (Fig 2B).

### Pathological changes in rats

#### (1) Gross morphology and light microscopy observations of H-E staining

Morphological changes in cells were observed by H-E staining. Compared with untreated rats and controls (Fig 2A and 2D), OA (0.06 mL/kg) induced a significant increase in histological lung damage (Fig 2B and 2C). The main histological changes seen via H-E staining included the presence of fibrin in the alveolar space, hemorrhage and necrosis of alveolar tissues, prominent edema, and neutrophil infiltration (Fig 2E). Whereas alveolar fibrin, hemorrhage, and necrosis were barely detectable in sections from the lungs of PS-treated animals (Fig 2F), all these features were significantly (*P* < 0.05) increased in lung sections from OA-PE rats.

#### (2) Electron microscopy observation of pulmonary surfactant layer (PSL) and vascular endothelial cells

**Fig 3:**
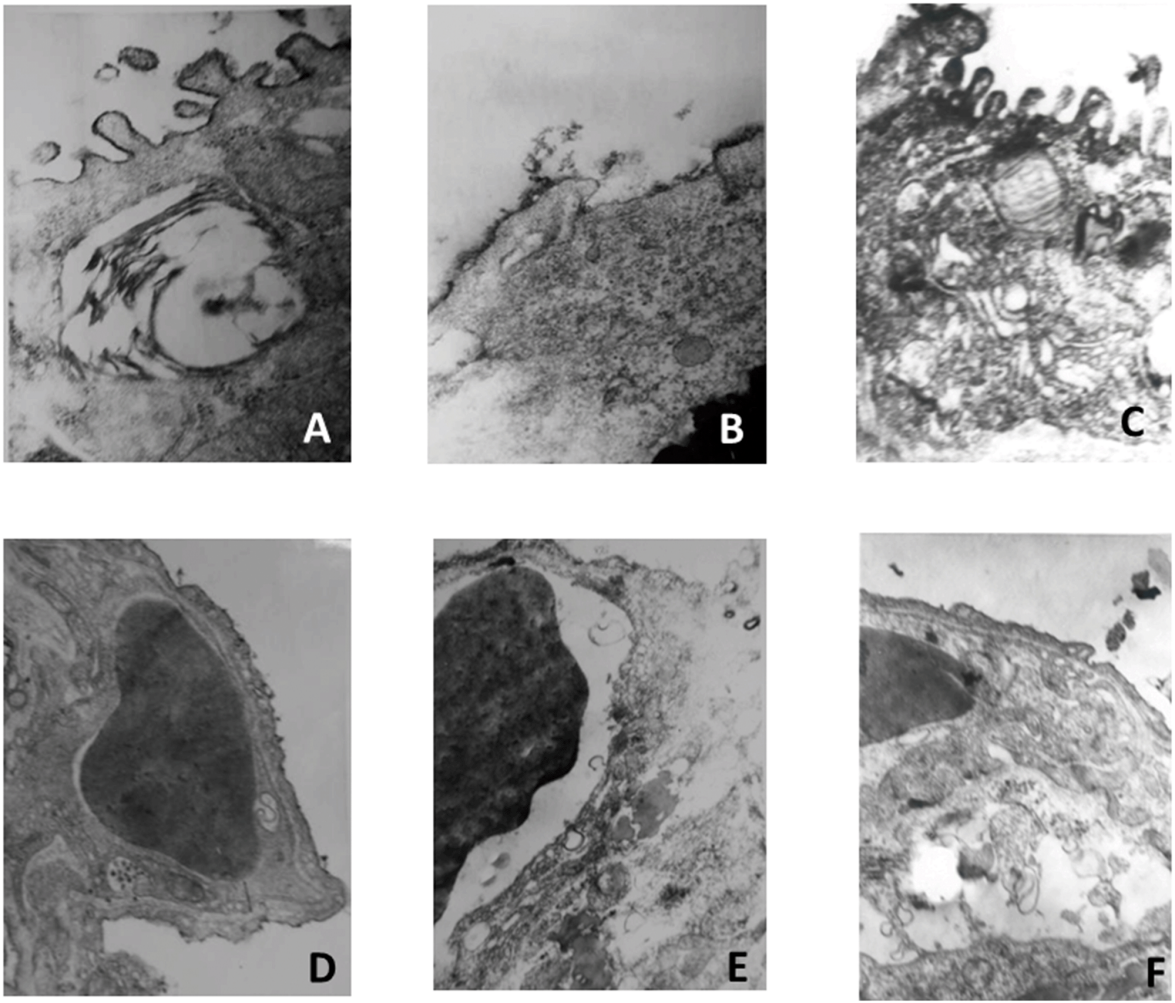
Electron microscopy observations of pulmonary surfactant layer (PSL) and vascular endothelial cells. A and D: Normal control group, B and E: OA-PE group, C and F: OA-PS treatment group. A, B, C: PSL; D, E, F: vascular endothelial cells.

Results from electron microscopy observations of the PSL (Fig 3A, 3B and 3C) and vascular endothelial cells (Fig 3D, 3E and 3F) indicate extensive loss of alveolar architecture following OA treatment. Large fragments of alveolar epithelial cells were detached from the basement membrane, whereas capillaries were grossly enlarged and showed some necrotic endothelial cells and denudation of both the epithelial and endothelial basement membranes. The PSL began to dissolve, and a microvascular endothelial cell layer and swelling of endothelial cells was apparent in the OA-induced PE group (Fig 3B and 3E), whereas an improvement in PSL and vascular endothelial cell structure was seen in the PS treatment group (Fig 3C and 3F). In the OA-PS treatment group, the aeration of alveoli was significantly improved compared with that in the OA-PE and control groups, as reflected by desquamation and necrosis of bronchiole epithelial cells. Photomicrographs representative of normal lungs without OA injury were also taken, and these cells exhibited normal bronchiole epithelial structure (Fig 3A and 3D).

As shown by the lung injury scores in Table 1, prominent edemas, infiltration of neutrophils, and intrapulmonary hemorrhages were observed in the lungs of animals in the OA-PE and OA-PS treatment groups. In the OA-PS treatment group, aeration of alveoli was moderately improved, significantly alleviating edema, hemorrhage, and bronchiole and alveolar epithelial desquamation compared to the OA-PE group (OA-PE: 9.1 ± 0.9; OA-PS: 4.8 ± 1.1, P < 0.05).

**Table 1:** Lung injury scores (LIS) in the three experimental groups.

Group	n	LIS
Control Group	6	0
OA-PE Group	6	9.1 ± 0.9 *
OA-PS Group	6	4.8 ± 1.1*

*compared with the other two groups, *P* < 0.05

### Surfactant phospholipid analysis in LT and BALF

OA-induced changes to TPL and DSPC in LT were assessed over time (4, 8, and 16 h) in the three groups (Table 2). Results show that TPL, DSPC, and the ratio of DSPC/TPL in LT decreased 4 h after being challenged with OA (0.06 mL/kg), but recovered at 8 and 16 h (values between groups were significantly different at 4, 8, and 16 h, *P* < 0.05). However, no significant difference could be detected between the OA-PE and control group regarding TPL, DSPC, or DSPC/TPL in BALF, as shown in Table 3.

**Table 2:** Changes in TPL and DSPC in lung tissue.

Group (n = 8)	TPL (mg/kg)	DSP (mg/kg)	DSPC/TPL (%)	TP (mg/kg)	DSPC/TP (%)
Control	9.5 ± 0.5	2.9 ± 0.5	30.9 ± 6.0	14.3 ± 2.5	21.4 ± 6.9
OA-4 h	6.8 ± 2.2*	1.4 ± 0.5*	22.8 ± 10.4	40.9 ± 11.2*	3.5 ± 1.2*
OA-8 h	10.7 ± 2.2	2.9 ± 0.3	28.2 ± 5.4	33.2 ± 13.5*	10.4 ± 4.7*
OA-16 h	9.1 ± 2.3	2.8 ± 0.4	32.9 ± 11.3	25.0 ± 4.6*	11.5 ± 2.6*

* *P* < 0.05 vs. control group

**Table 3:** Changes in TPL and DSPC in BALF.

Group (n = 8)	TPL (mg/kg)	DSPC (mg/kg)	DSPC/TPL (%)
Control	100.0 ± 6.1	18.2 ± 5.1	18.1 ± 4.2
OA-4 h	104.0 ± 12.3	18.0 ± 4.1	17.5 ± 4.0
OA-8 h	108.4 ± 11.4	25.1 ± 5.2*	23.2 ± 4.7*
OA-16 h	122.0 ± 22.1	23.4 ± 3.3*	19.6 ± 3.6

* *P* < 0.05 vs. control group

### Radiolabel analysis of LT and BALF

**Fig 4:**
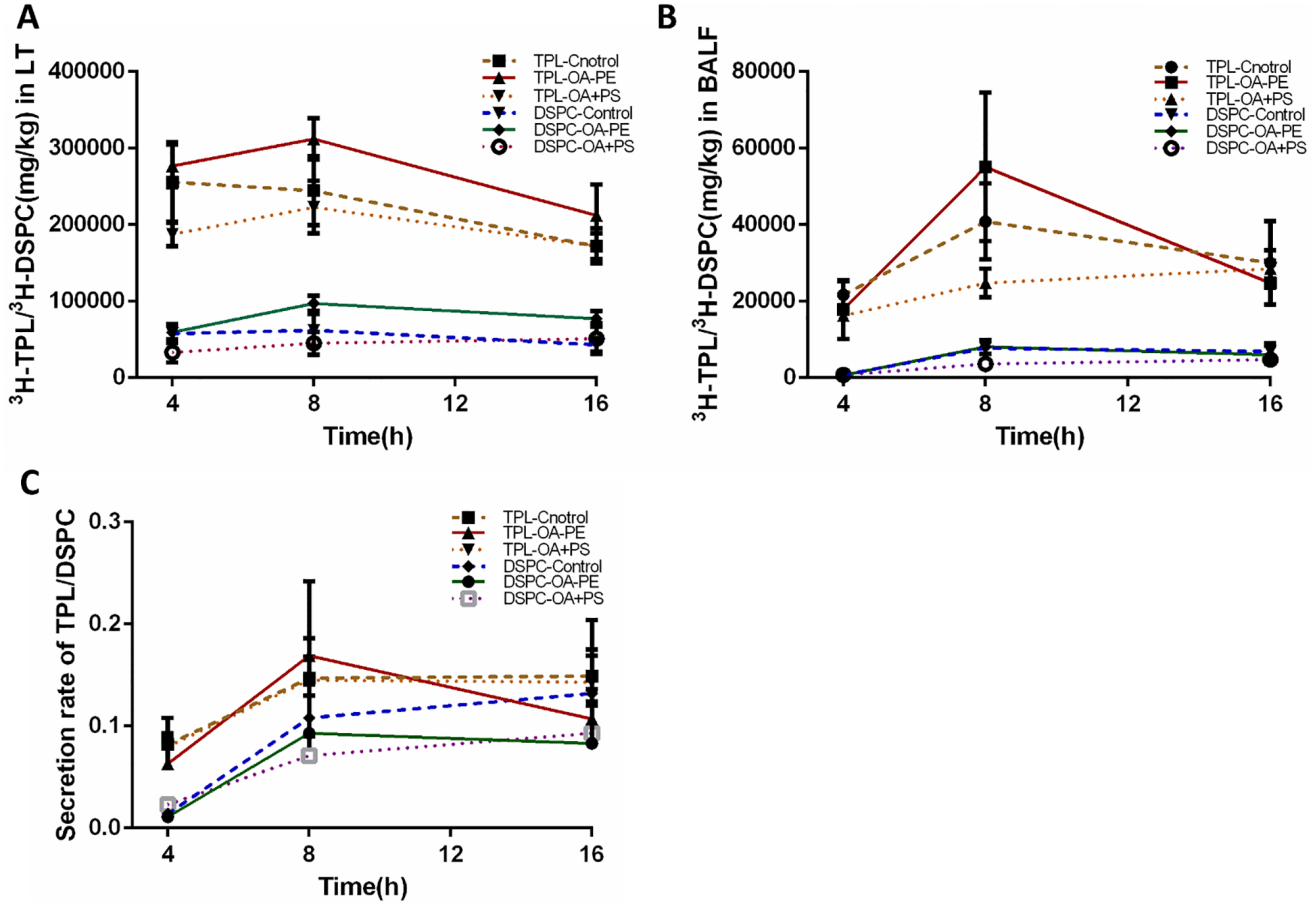
Changes in ^3^H-TPL and ^3^H-DSPC levels in LT and BALF. A: Changes in ^3^H-TPL and ^3^H-DSPC levels in LT. B: Changes in ^3^H-TPL and ^3^H-DSPC levels in BALF. C: Changes in TPL SR and DSPC SR.

Changes in ^3^H-TPL and ^3^H-DSPC in LT and BALF samples between the three groups over time (4, 8, 16 h) are shown in Fig 4. Accordingly, 8 h after injection of OA, radioactivity of ^3^H-TPL and ^3^H-DSPC in the lungs reached its peak, with CPM of ^3^H-DSPC being significantly lower in the PS treatment group (LT: Control: 62327 ± 9108; OA-PE: 97315 ± 10083; OA-PS: 45127 ± 10034, P < 0.05; BALF: Control: 7771 ± 1768; OA-PE: 8097 ± 1799; OA-PE: 3651 ± 1027, P < 0.05). There was no difference between the three groups in terms of ^3^H-TPL in LT or BALF at any time point. Furthermore, DSPC SR in the lungs was significantly lower in the PS treatment group at 4 h after injection (Control: 0.014 ± 0.003; OA-PE: 0.011 ± 0.004; OA-PS: 0.023 ± 0.006, P < 0.05), as shown in Fig 4 and in S1 and S2 Tables.

## Discussion

PS is made up of several classes of lipids, including phospholipids, triglycerides, cholesterol, and fatty acids; but may contain also the surfactant proteins SP-A, SP-B, SP-C, and SP-D. PS is the key treatment for ARDS, and its administration is considered effective and safe [1]. However, the complex pathology and underlying mechanisms of ARDS have so far prevented PS replacement therapy from improving mortality in ARDS. Replacement therapy for ALI preserves the functional surfactant phospholipid pool size and it may lessen the severity of lung injury during sepsis or in animal models [11]. As replacement becomes more successful, it will be important to determine how PS affects the regulation of PC synthesis.

The *de novo* PS synthesis pathway depends largely on the availability of PC in type II cells. Studies have confirmed that OA can induce a series of damages causing extensive loss in alveolar architecture [14]. Large fragments of alveolar epithelial cells that detach from the basement membrane are grossly enlarged, and the swelling of endothelial cells is accompanied by initial PSL dissolution. Consistent with this findings, the endogenous synthesis of PS was not improved in our study even though an improvement in PSL and vascular endothelial cell structure was observed in the PS treatment group.

In our animal model of OA-induced PE treated or untreated with PS, we mimicked the metabolism of PS and determined the effect of exogenous PS treatment on its synthesis. Samples taken at 4, 8, and 16 h allowed the assessment of time-dependent changes in PS synthesis in LT and BALF. We found that OA injection profoundly disrupted both the endothelium and epithelium of the lungs, resulting in acute increases of total alveolar protein (TP) in LT and TPL in BALF. At the same time, a marked deteriorations in DSPC and TPL in LT and the DSPC/TPL ratio in BALF was detected at 4 h, despite a recovery at 8 and 16 h, compared with the control group (*P* < 0.05). In fact, blood contains very few free fatty acids. However, normalized alveolar lung water and TPL or DSPC levels at 16 h did not suggest that the DSPC/TP ratio returned to normal levels. Paradoxically, the total alveolar DSPC pool increased after PS treatment. According to radiolabel analysis of LT and BALF, our findings illustrate that OA dramatically increased the levels of ^3^H-TPL and ^3^H-DSPC in BALF at 8 h in OA-induced PE compared with those in controls, while markedly reducing the DSPC/TPL ratio during the first 4 h.

PC has been shown to originate from free fatty acids in circulation or triacyclglycerols within lipoproteins [15]. It is not surprising that the activities and levels of PC are insufficient to ameliorate the injury induced by OA during the first 4 h. OA may downregulate the mRNA levels of key enzymes involved in PC synthesis, such as members of the choline kinase (CK) family, which catalyze the phosphorylation of choline into choline-phosphate, the first committed step in PC synthesis [16]. In mammals, the CK family contains CKα and CKβ, which are encoded by two different genes. CTP: phosphocholine cytidylyltransferase (CCT) is the predominant key enzyme in the lungs and is extensively regulated by both activating and inhibitory lipids [17]. Choline phosphotransferase (CPT) plays an important role in regulating the acyl group composition of PC in the lungs and other mammalian tissues [18]. The final step in the *de novo* synthesis of PC is catalyzed by CPT, a membrane-bound enzyme localized primarily in the endoplasmic reticulum. CPT plays an important role in regulating the acyl group composition of PC in the lungs and is dependent on CDP-choline availability [19]. This enzyme is able to utilize both endogenous and exogenous diacylglycerol for PC synthesis in the lungs [20].

Hormonal or physiological factors can also stimulate *de novo* fatty acid synthesis by increasing the immunoreaction levels of fatty acid synthase (FAS) [21]. The number of sites of phosphatidic acid (PA) biosynthesis could be related to access to specific substrates, which is linked to the eventual newly synthesized phospholipids or the cell’s needs [22]. Therefore, the elevated level of PC at 4 h may reflect a feedback mechanism for reducing the injury caused by OA, whereas the reduction in PC synthesis in the PS treatment group at 16 h may reflect feedback inhibition based on the cell’s requirements.

In 1993, Ikegami et al. [21] found that surfactant treatments had two primary effects on preterm lungs: one was a physiological effect related to surface properties, and the other was a metabolic effect resulting from the exogenous surfactant phospholipid functioning as a substrate for recycling pathways. This was based on metabolic studies of surfactant lipids in term lambs. However, the study showed that surfactant treatment did not adversely affect endogenous synthesis through feedback inhibition. Recent studies [23] have demonstrated that anionic phospholipids play an important role in altering the immune response during viral infection. If the proper balance between physiological regulators is disturbed or inhibitory factors are released, as during lung inflammation, surfactant deficiency can significantly contribute to impairment of lung mechanics. During both acute and chronic respiratory illness, PS levels can be reduced. A study shows that ALI can set in motion a sequence of events that increase respiratory failure irrespective of the size of the surfactant pool and the integrity of the alveolocapillary barrier [6]. These interactions regulate the structure and properties of the secretory rate of DSPC and nonspecific immune response, which could be another possible explanation for the acute lung injury observed in our model. Nevertheless, the secretory mechanisms of pulmonary surfactant are still not fully characterized. Much of our molecular and physiological understanding of choline kinases in relation to surfactant metabolism remains to be determined.

## Conclusions

In summary, the present study developed a new method to assess changes in the synthesis of PC and to determine the effect of exogenous PS on its *de novo* synthesis in OA-induced PE rats. The radioactivity of ^3^H incorporated into TPL and DSPC was measured in BALF and LT samples. According to the radiolabels, expressed as CPM, in the OA-induced PE model and OA-PS treatment group, we hypothesize that exogenous PS treatment may adversely affect endogenous *de novo* synthetic and secretory phospholipid pathways by feedback inhibition. However, this mechanism may not be straightforward, and additional research is therefore needed.

## Supporting information

S1 Fig Ethical statement. (JPG)

S1 Table Changes in ^3^H-TPL and ^3^H-DSPC in LT and BALF. (DOCX)

S2 Table Changes in TPL SR and DSPC SR. (DOCX)
