# Supplementary figures and images for: Synthesis of phosphatidylcholine in rats with oleic acid-induced pulmonary edema and effect of exogenous pulmonary surfactant on its De Novo synthesis
Source: PLoS One. 2018 Mar 19;13(3):e0193719. doi: 10.1371/journal.pone.0193719 (PMC5858825; doi:10.1371/journal.pone.0193719)

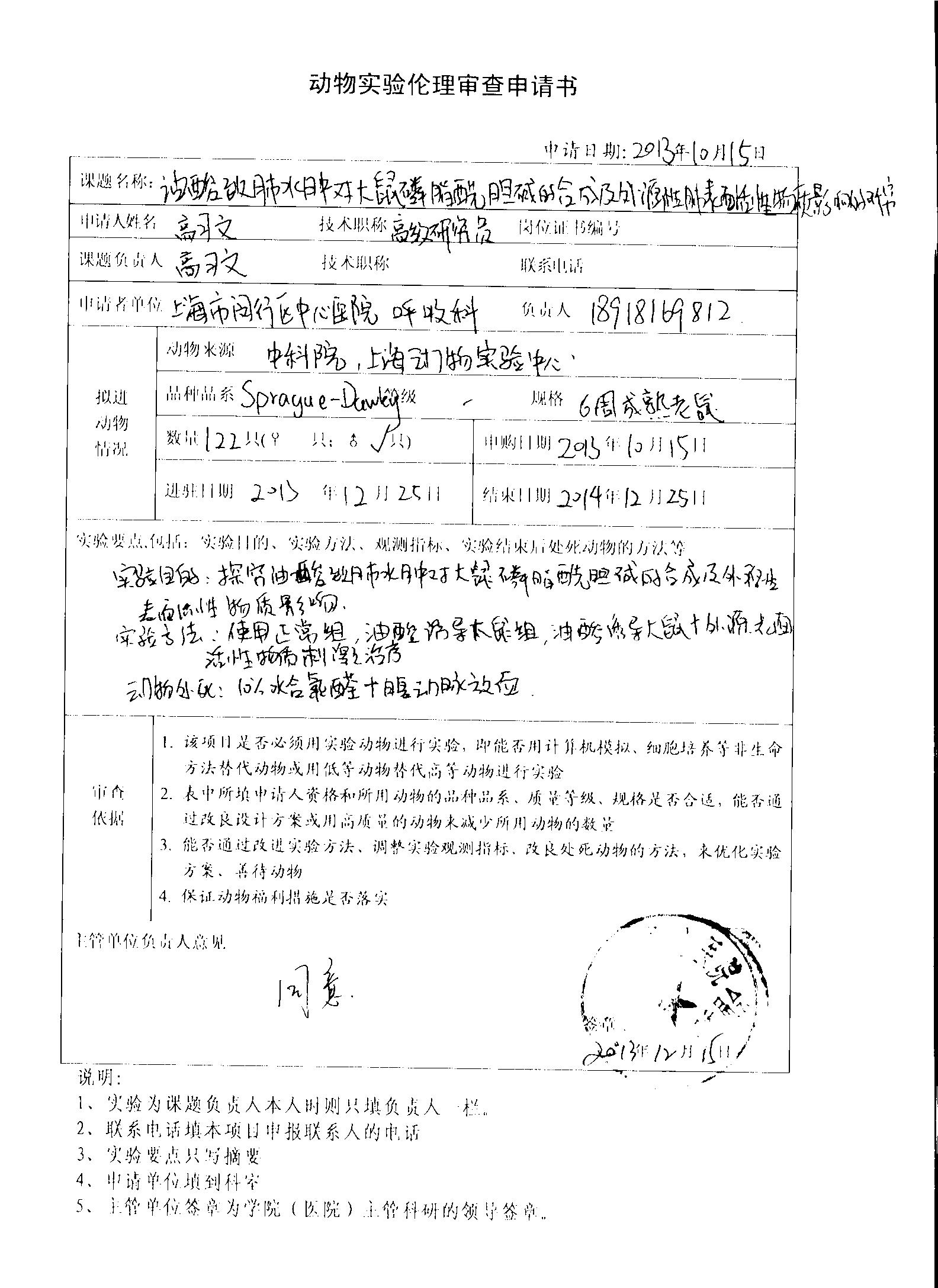

Supplement: S1 Fig — (JPG) [file pone.0193719.s001.jpg]
