# Supplementary material for: Synthesis of phosphatidylcholine in rats with oleic acid-induced pulmonary edema and effect of exogenous pulmonary surfactant on its De Novo synthesis
Source: PLoS One. 2018 Mar 19;13(3):e0193719. doi: 10.1371/journal.pone.0193719 (PMC5858825; doi:10.1371/journal.pone.0193719)
Supplement: S1 Table — (DOCX) [file pone.0193719.s002.docx]

**Table S1. Changes in ^3^H-TPL and ^3^H-DSPC in lung tissue and BALF.**

| Groups | Lung tissue | | | | | | BALF | | | | | | |
| --- | --- | --- | --- | --- | --- | --- | --- | --- | --- | --- | --- | --- | --- |
|  | ^3^H-TPL (CPM) | | | ^3^H-DSPC (CPM) | | | ^3^H-TPL (CPM) | | | ^3^H- DSPC (CPM) | | | |
|  | 4 h | 8 h | 16 h | 4 h | 8 h | 16 h | 4 h | 8 h | 16 h | 4 h | 8 h | 16 h |  |
| Control | 255854 ± 51947 | 244364 ± 44837 | 171939 ± 16449 | 57829 ± 12339 | 62327 ± 9108 | 43002 ± 13325 | 21703 ± 3551 | 40853 ± 9973 | 30049 ± 10856 | 799 ± 93 | 7771 ± 1768 | 6899 ± 2146 |  |
| OA - PE | 276755 ± 28186 | 312324 ± 27267 | 212149 ± 40794 | 59336 ± 20748 | 97315 ± 10083# | 77024 ± 15098 | 17818 ± 7657 | 55101 ± 19431 | 24770 ± 5707 | 670 ± 140* | 8097 ± 1799 | 6018 ± 2332* |  |
| OA + PS | 187442 ± 15372# | 222808 ± 34241 | 172234 ± 22880 | 33256 ± 8901# | 45127 ± 10034# | 51126 ± 19875 | 16223 ± 1029* | 37279 ± 3717 | 28474 ± 4867 | 766 ± 121 | 3651 ± 1027# | 4745 ± 928* |  |

*----Compared with control group, P < 0.05

#----- Compared with the other two group, P < 0.05
