# Supplementary material for: Synthesis of phosphatidylcholine in rats with oleic acid-induced pulmonary edema and effect of exogenous pulmonary surfactant on its De Novo synthesis
Source: PLoS One. 2018 Mar 19;13(3):e0193719. doi: 10.1371/journal.pone.0193719 (PMC5858825; doi:10.1371/journal.pone.0193719)
Supplement: S2 Table — (DOCX) [file pone.0193719.s003.docx]

**Table S2. Changes in TPL SR and DSPC SR.**

| Groups | TPL SR* | | | DSPC SR* | | |
| --- | --- | --- | --- | --- | --- | --- |
|  | 4 h | 8 h | 16 h | 4 h | 8 h | 16 h |
| Control | 0.082 ± 0.026 | 0.147 ± 0.039 | 0.149 ± 0.055 | 0.014 ± 0.003 | 0.108 ± 0.022 | 0.132 ± 0.043 |
| OA - PE | 0.063 ± 0.030 | 0.169 ± 0.073 | 0.107 ± 0.029 | 0.011 ± 0.004 | 0.093 ± 0.048 | 0.083 ± 0.037 |
| OA + PS | 0.080 ± 0.010 | 0.145 ± 0.023^@^ | 0.143 ± 0.026 | 0.023 ± 0.006^#^ | 0.071 ± 0.019* | 0.093 ± 0.031 |

^@^——Compared with OA-PE group，P < 0.05

*----Compared with control group, P < 0.05

^#^----Compared with the other two group, P < 0.05

*TPL SR(secretion rate)＝TPL-^3^H/total phospholipid（TPL）harvested in 4h, 8h and 16h after OA infusion.

*DSPC SR(secretion rate)＝DSPC-^3^H /TPL in 4h, 8h and 16h after OA infusion.
